# Prophylactic antibiotic for prevention of posttraumatic meningitis after traumatic pneumocephalus: design and rationale of a placebo-controlled randomized multicenter trial [ISRCTN71132784]

**DOI:** 10.1186/1745-6215-7-2

**Published:** 2006-01-18

**Authors:** Behzad Eftekhar, Mohammad Ghodsi, Azar Hadadi, Mousa Taghipoor, Samira Zabihyan Sigarchi, Vafa Rahimi-Movaghar, Ehsan Sherafat Kazemzadeh, Babak Esmaeeli, Farideh Nejat, Alireza Yalda, Ebrahim Ketabchi

**Affiliations:** 1 Sina Trauma and Surgery Research Center and Department of Neurosurgery, Sina Hospital, Tehran University, Iran; 2 Department of Infectious Diseases, Sina Hospital, Tehran University, Iran; 3 Department of Neurosurgery, Namazi Hospital, Shiraz University, Iran; 4 Department of Neurosurgery, Ghaem Hospital, Mashad University, Iran; 5 Department of Neurosurgery, Khatam-ol-anbia Hospital, Zahedan University, Iran; 6 Department of Infectious Diseases, Imam Hospital, Tehran University, Iran; 7 Department of Neurosurgery, Hospital, Shahrood, Iran; 8 Department of Neurosurgery, Children's Hospital Medical Center, Tehran University, Iran

## Abstract

**Background:**

The purpose of this study is to compare the efficacy of prophylactic antibiotic for prevention of meningitis in acute traumatic pneumocephalus patients.

**Methods:**

In this prospective, randomized controlled clinical trial, 200 selected head injury patients with traumatic pneumocephalus are randomly assigned to receive intravenous antibiotics (2 grams Ceftriaxone twice a day), oral antibiotics (Azithromycin) or placebo for at least 7 days after trauma. The patients will be followed for one month posttrauma.

**Conclusion:**

The authors hope that this study helps clarifying the effectiveness and indications of antibiotics in prevention of meningitis in traumatic pneumocephalus after head injury and in specific subgroup of these patients.

## Background

Chemoprophylaxis with antibiotics is both feasible and desirable for prevention of a potentially serious disease when specific groups at risk can be defined and when a safe, effective, and affordable prophylactic agent is available [1]. One of such potentially serious diseases is posttraumatic meningitis. The incidence of posttraumatic meningitis after head trauma ranges from 0.2 to 17.8 per cent and increases significantly in the presence of skull base fracture, pneumocephalus or cerebrospinal fluid (CSF) leak [2,3].

Considering the serious complications of the posttraumatic meningitis, the idea of chemoprophylaxis with antibiotics for prevention of posttraumatic meningitis has always been considered rational, but the efficacy of prophylactic antibiotic agents in the setting of posttraumatic CSF leakage is still controversial. The contrasting findings reported by two large studies in which metaanalyses were conducted reflect the general disagreement and lack of sufficient power of the previous studies [4-6].

One possible reason for the inability of the previous studies to demonstrate the efficacy of prophylactic antibiotics for prevention of posttraumatic meningitis might have been inconsideration of those subsets of patients with specific risk factors [5].

With this concept in mind, the authors tried to clarify those subgroups at risk in two subsequent studies since 1994. In the first study done in our hospital (Ghodsi M, Nejat F, Eftekhar B, unpublished data), the efficacy of prophylactic antibiotics in head injury patients with skull base fracture were studied. Although no statistically significant differences were found between those with and without prophylactic antibiotics, 12% of patients with skull base fractures had pneumocephalus and the incidence of meningitis was considerably higher (20%) in this subgroup of the head injury patients. In the second study we selected only patients with posttraumatic pneumocephalus. The overall meningitis rate in head injury patients with pneumocephalus was 21.5% [5]. That study again did not approve the prophylactic administration of ceftriaxone (2 grams daily) in the pneumocephalus patients. But it showed that CSF rhinorrhea and intracranial hemorrhage might be considered as primary risk factors and in the absence of them, intradural location of air and air volume greater than 10 cc as secondary risk factors for developing meningitis in posttraumatic pneumocephalus patients [5]. Even in those patients with CSF rhinorrhea or intracranial hemorrhage, prophylactic ceftriaxone (2 grams daily) did not prove to be effective on prevention of meningitis.

### Selection of drugs

In posttraumatic meningitis after traumatic pneumocephalus, the microorganisms enter the CSF from the outside mainly air sinuses. This is the reason why pneumococci are the major causative microorganisms. In order to prevent posttraumatic meningitis in pneumocephalus patients, we should either clear the air sinuses from the potential microorganisms or make the CSF defense strong enough to control those pathogenic microorganisms that have passed the blood brain barrier. This simple strategy has not worked favorably so far. There might be various reasons. We thought that the dosage of the antibiotics might have been less than enough due to lack of inflammation of the blood brain barrier. So based on the most common microorganisms in microbiological studies of the posttraumatic meningitis it has been decided to administer antibiotics with full antimeningitic dosage (2 grams Ceftriaxone twice a day) prophylactically.

Another reason for inability of the previous studies to demonstrate the efficacy of prophylactic antibiotics might have been lack of enough coverage of the normal flora of the air sinuses. These sinuses should be considered the potential source of the secondary infection in majority of cases. In order to cover these, we selected Azithromycin as an oral regimen. Penetration of drugs into the cerebrospinal fluid, minimal gastrointestinal side effects, patient tolerance and cost-effectiveness of medications were other important factors in selection of drugs.

In our previous studies meningitis happened almost always in the first week post trauma, so both regimens are continued for 7 days.

### Objectives

#### Primary objective

The primary objective of this prospective study is to compare the efficacy of prophylactic antibiotics (either intravenous Ceftriaxone 2 grams BID or oral Azithromycin) for prevention of bacterial meningitis in acute traumatic pneumocephalus.

#### Secondary objectives

- To determine the relative frequency of rhinorrhea and intracranial hemorrhage in each of the IV, O and P groups

- To determine the GCS, age and volume of intracranial air in each of the IV, O and P groups

- To determine if the volume or location of the air affects the rate of meningitis.

- To determine the rate of presence and location of the skull base fracture in each of the IV, O and P groups

- To determine the short term (during the treatment) complications of the administration of the antibiotics in each of the IV, O and P groups

- To compare the microbiological findings in meningitic patients among IV, O and P groups

- To determine the frequency of meningitis caused by resistant bacteria.

### Outcome measures

#### Primary outcome measure

The frequency of bacterial meningitis in IV, O and P groups

#### Secondary outcome measures

- The frequency of rhinorrhea, intracranial hemorrhage and skull base fracture, volume and location of intracranial air in the population study and each of the IV, O and P groups.

- The mortality rate in study population and each of the IV, O and P groups.

## Methods

### Study Population

The study is intended to be a randomized placebo-controlled clinical trial study.

### Sample size

The frequency rate of meningitis among our traumatic pneumocephalus patients was 21.5% (*p*1). What we expect to be an intended (or at least acceptable) effect of the prophylactic antibiotics is 5 %(*p*2) frequency of meningitis in the patients with antibiotics. To detect this difference with a sensitivity of 80% and an error probability of 5%, at least 62 patients per randomization group will be required using the following formula:

*n *= 7.84 * [*p*1(1 - *p*1) + *p*2 (1 - *p*2)]/[*p*_1 _- *p*_2_]^2^

Considering only comparing the placebo group with each one of the antibiotics group, our sample size should be at least 186 cases. To account for the possibility of loss to follow-up, our estimated sample size is 200 cases.

### Ethical approval

This study has been ethically approved by Sina Trauma and Surgery Research Center, Tehran University.

### Inclusion criteria

The entry criterion to this study is traumatic pneumocephalus verified by brain CT scan. The patients should be older than 15 years old, admitted less than 24 hours after trauma and we should be able to start the antibiotics in less than 24 hours after trauma.

### Exclusion criteria

Those patients who have received antibiotic therapy for other reasons within the prior 2 weeks, are on corticosteroids or allergic to the specified medications, individuals with penetrating traumatic brain injury, open skull fractures or operated for any causes, those who are discharged from hospital with personal consent, all cases with life threatening lesions including severe brain, abdominal or vascular injuries and death due to other causes will be excluded from this study.

### Randomization

The randomization list was generated by using the website Randomization.com . The permuted block method of randomization for a block size of three was used. According to this list patients were allocated to each of the three groups in the trial: intravenous antibiotics, oral antibiotics and placebo.

### Blinding

Since providing both the antibiotics and placebo in identical containers for participating centers is not practically possible for us, this study is not double-blinded.

### Study design

The patients should be observed in the hospital, until occurrence of meningitis or at least 7 days after trauma. In the case of concomitant Cerebrospinal Fluid (CSF) leakage and need for close observation in the hospital, they will be hospitalized until recovery from leakage. After discharge the patients will be followed up until one month after trauma.

The following data is registered: age, sex, time interval between trauma and admission, time interval between admission and antibiotic therapy, the cause of trauma, GCS upon admission, intracranial air volume (at the time of admission and three days later), intracranial air location, presence of CSF rhinorrhea, CSF otorrhea, presence and location of the radiological signs of skull base fractures, presence and volume of the intracranial hemorrhage(at the time of admission and three days later), presence of meningitis, CSF findings, treatment and complications in the case of meningitis and one month follow-up note.

Intracranial air and hemorrhage volume are calculated in the CT scan using the formula *ABC*/2, where *A *is the greatest mass diameter by CT, *B *is the diameter 90° to *A*, and *C *is the approximate number of CT slices with mass multiplied by the slice thickness. In the case of air in the subarachnoid space, this formula is not easily applicable and an approximate volume is calculated and determined whether the volume is less than 10 cc or not [7].

The patients are divided into three groups; with intravenous antibiotics (IV), oral antibiotics (O) and placebo (P), according to the randomization list. In IV group, Ceftriaxone 2 grams BID plus oral placebo and in O group, Azithromycin 500 mg in the first day followed by 250 mg daily plus intravenous placebo for the rest will be continued for 7 days. Antibiotics should be started in less than 24 hours after trauma. Since antibiotics may mask the clinical presentation of the meningitis, these patients (IV and O) will remain hospitalized two more days for close clinical observation. In the case of meningitis, appropriate antibiotics are continued until patients' recovery. Corticosteroids should not be prescribed for the patients for any reason.

The physicians caring for the patients are not blinded to the regimen that is used.

Intracranial hemorrhages included subarachnoid hemorrhage, epidural and subdural hematoma and cerebral contusion or hematoma.

The diagnosis of bacterial meningitis is based upon CSF findings (increase in leukocyte count, decrease in CSF glucose [<60% of the level simultaneously measured in blood] increase in the CSF protein > 45 mg/dL and positive CSF smear or culture) in patients with compatible clinical findings (fever, headache, nausea and vomiting, change in the level of consciousness and/or meningismus not explainable with other causes). Probable meningitis is defined when positive CSF smear or culture is not available. In rare cases with relative contraindications for lumbar puncture (accompanying intracranial hemorrhage and clinically suspected raised intracranial pressure), the possible diagnosis of meningitis is based upon clinical findings and ruling out other causes.

### Statistical analysis

Intention to treat analysis will be used.

Categorical variables are compared using a chi-square test, and the Student t-test is used to compare continuous variables between groups. Logistic regression with adjustment for other possible risk factors for meningitis is used to estimate the odds ratios and 95% confidence intervals.

## Discussion

This study can be a major step forward solving one of the controversial issues in the management of traumatic pneumocephalus. Considering our circumstances the design of the study is not double-blinded. This may be one of the potential shortcomings of the study.

## Conclusion

In conclusion, this study is aimed to study the effectiveness and indications of prophylactic antibiotics in prevention of meningitis in traumatic pneumocephalus patients after mild head injury.

## Competing interests

The author(s) declare that they have no competing interests.

## Authors' contributions

BE conceived the trial design, involved in subsequent adaptations and drafted the manuscript. MG, AH MT, SZS, VRM, ESK, BE, FN, AY and EK have contributed to adaptations from the original design.

All authors read and approved the final manuscript.
